# Growth Hormone Deficiency and Diabetes Insipidus as a Complication of Endoscopic Third Ventriculostomy

**DOI:** 10.4274/Jcrpe.801

**Published:** 2012-12-19

**Authors:** Kimberly S. Tafuri, Thomas A. Wilson

**Affiliations:** 1 Stony Brook University, Department of Pediatrics, Stony Brook Childrens Hospital, Division of Pediatric Endocrinology, Stony Brook, NY

**Keywords:** Polyuria, triphasic pattern, child, Growth hormone deficiency, acute cerebellitis

## Abstract

Endoscopic third ventriculostomy (ETV) has become the procedure of choice for the treatment of obstructive hydrocephalus in children and adults. Endocrinological complications of ETV in children are rare. Diabetes insipidus (DI) is the most common and accounts for only 0.5% of complications from ETV. The majority of documented cases are transient. To date, there are no documented cases of multiple pituitary hormone deficiencies. We present here a 6-year-old girl with growth hormone deficiency and permanent DI which developed as a complication of ETV. This patient is unique in both demonstrating multiple pituitary hormone deficiencies and the classical triphasic response of DI after ETV. We postulate that these complications were caused by compression of the pituitary stalk and hypothalamic injury during the procedure. We compare our case presentation to experimental studies conducted in rats.

**Conflict of interest:**None declared.

## INTRODUCTION

Endoscopic third ventriculostomy (ETV) is the procedure of choice for the treatment of obstructive hydrocephalus. Surgical intervention for hydrocephalus associated with acute cerebellitis is less common. Surgical procedures such as posterior fossa decompression and external ventricular drainage have also been reported (1). Endocrinological complications of ETV in children are rare. Diabetes insipidus (DI) is the most common and accounts for only 0.5% of complications from ETV (2). Two large retrospective cohort studies of children undergoing ETV did not show any cases of central DI (3,4). The majority of documented DI cases following ETV are transient. To our knowledge, 3 cases of permanent DI have been reported, none of which presented with a triphasic response (5,6,7). We present the only reported case of a child with permanent DI demonstrating a classic triphasic response of DI after ETV.

Currently, there are no documented cases of patients who sustained multiple pituitary hormone deficiencies as a complication of ETV and no reported cases of isolated growth hormone (GH) deficiency. The finding of GH deficiency in our patient makes this case even more unique.

## CASE

A 6-year-old previously healthy girl presented with fever, myalgia, intermittent diploplia and progressive headache associated with non-bilious emesis. The symptoms had started in the past 3 days. The patient was brought to the emergency clinic with intractable pain from worsening headaches and acute onset of “staggering gait”. Initial vital signs were normal. Physical examination was normal except for the appearance of the patient as a tired, anxious child and presence of bilaterally purulent tympanic membranes. A baseline computed tomography (CT) scan of the head was obtained which revealed bilateral maxillary sinus disease. She was admitted to the hospital for intravenous hydration and antibiotics.

The headaches worsened during the following 24-48 hours. A magnetic resonance imaging/magnetic resonance angiography revealed obstructive hydrocephalus, diffuse bilateral cerebellar swelling and a normal-appearing pituitary gland. Surgical decompression via ETV with external ventricular drain placement was carried out with resolution of the patient’s headaches. Pre-operatively the patient’s serum sodium concentration and urine output were normal. Postoperatively, the patient developed polyuria and polydipsia with a urine output of 9.8 mL/kg/hr, leading to a negative balance in body water, mild hypernatremia and elevated serum osmolality. Urine osmolality was low with an undetectable arginine vasopressin (AVP) level consistent with central DI (Table 1). Oral desmopressin was administered with improvement in urine output to 3.08 mL/kg/hr.

**Figure 1:**
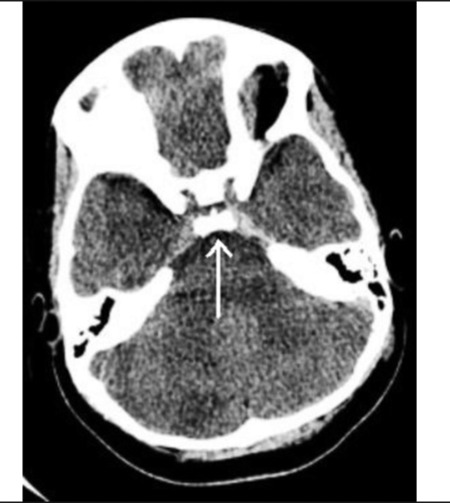
Computed tomography (CT) scan of brain on post-operativeday 1 demonstrating the catheter tip just above the level of the sellaturcica. Arrow points to catheter tip

Post-operative CT scan revealed the catheter tip in the floor of the third ventricle (Figure 1). The catheter tip was retracted, but the desmopressin requirement persisted until late post-operative day 2. The last dose of desmopressin was administered in the evening of post-operative day 2. On post-operative day 3, serum sodium concentration was normal (Table 1), and urine output was 1.9 mL/kg/hr without desmopressin. Screening tests of anterior pituitary function were normal (Table 2). By post-operative day 6, the urine output had reached stability and urine osmolality was 622 mOsm/kg. The hydrocephalus eventually improved. The ventricular drain was removed and the child was discharged home without desmopressin. No etiology for the cerebellitis was identified. CSF studies, lyme serology, viral, fungal and bacterial cultures were negative.On post-operative day 10, the child again developed polyuria and polydipsia. Laboratory evaluation was consistent with recurrence of central DI (Table 1). She was restarted on desmopressin.

One year after surgery, the patient was found to have persistent central DI and a deceleration in growth velocity. Prior to the procedure, she was tracking along the 90th percentile in height with an annual growth velocity of approximately 7 cm and no clinical evidence of hypothalamic or pituitary dysfunction. Post ventriculostomy, her growth velocity progressively declined to 2.4 cm/yr over a 9-month observation period. Despite normal insulin-like growth factor (IGF-1) and IGF binding protein-3 (IGFBP-3) levels, a GH stimulation test with arginine and clonidine was performed which resulted in a peak GH level of 8.8 ng/mL, confirming the diagnosis of partial GH deficiency. GH therapy was recommended but has not yet been initiated. Currently, all other anterior pituitary hormones remain intact (Table 2).

## DISCUSSION

The triphasic response in the development of central DI is characterized by polyuria and dilute urine, followed by reduction in urine output and restoration of urine concentration, and finally, recurrence of polyuria and dilute urine. This response is thought to be secondary to early hypothalamic dysfunction of the AVP secreting neurons resulting in DI, subsequent release of AVP from the AVP nerve terminals in the degenerating pituitary gland resulting in temporary resolution of DI, and finally, depletion of all AVP stores with resultant permanent DI (8). The triphasic pattern is relatively uncommon (3.4%) even in patients undergoing transphenoidal surgery (8). Since DI as a complication of ETV is usually transient, the number of patients manifesting the classic triphasic response is unknown. Although our patient never developed hyponatremia, which is typical of inappropriate secretion of AVP, she clearly had transient recovery of posterior pituitary function as evidenced by her ability to concentrate her urine without desmopressin, thus demonstrating all three phases of the triphasic pattern.

In vivo studies in rats have demonstrated that various types of axonal injury to the hypothalamo-neurohypophyseal tract may lead to degeneration of the magnocellular neurons in the hypothalamus and development of central DI. Dohanics et al (9) elicited central DI with a classic triphasic pattern with controlled compression of the rat pituitary stalk. Gentle compression of the pituitary stalk, at the level of the magnocellular axons, for only 30 seconds, resulted in degeneration of the magnocellular nerve terminals of the posterior pituitary gland. Compression of the magnocellular axons led to the selective destruction of the posterior lobe with preservation of anterior lobe function (9). Selective damage to the posterior lobe with sparing of the anterior lobe has been well documented in rat studies through various surgical techniques (9,10).

Although endocrinological complications from ETV are rare, the proximity of the hypothalamus and pituitary stalk to the third ventricular floor makes these structures particularly vulnerable to injury. Given the loss of AVP function in our patient, we postulate that placement of the ventricular catheter into the third ventricle compressed the magnocellular neurons which resulted in degeneration of the AVP neurons, a mechanism similar to that reported in rat studies. Prior studies have demonstrated that destruction of greater than 80% to 90% of the AVP neurons is required before permanent DI ensues (8).

The co-occurrence of GH deficiency has not been previously reported. A prospective study of 20 children who underwent endocrine evaluation after ETV revealed one patient with a low IGF-1 and IGFBP-3 with normal height and growth velocity (11). Since the growth velocity was normal in this reported case, GH stimulation testing was not carried out. Our case demonstrates normal IGF-1 and IGFBP-3 with a subnormal growth velocity and a subnormal GH response to pharmacological stimulation, a pattern which has been reported by Haghshenas et al (12). Of the 81 short stature patients evaluated in this report, 17 had documented GH deficiency based on provocative stimulation testing, 6 of the 17 had both low GH and IGF-1, and 2 of the 17 had low GH and IGFBP-3, thus emphasizing the importance of auxological measurement and growth velocity (12). The observation of subnormal GH secretion coupled with subnormal growth velocity in our patient suggests that additional hypothalamic damage occurred. Hypothalamic damage rather than stalk compression could also account for the DI in this patient.

## CONCLUSION

We present this case to emphasize the importance of considering endocrinological complications after ETV. Permanent DI, although rare, can occur. The proximity of the hypothalamic structures to the third ventricular floor makes the hypothalamic nuclei and neurons susceptible to injury during perforation with a ventricular catheter (2). In the case described above, we speculate that advancement of the ventricular catheter led to temporary compression or injury of the hypothalamic nuclei and/or pituitary stalk sufficient to cause destruction of greater than 80% of the functional AVP neurons resulting in permanent DI with a classic triphasic pattern. Recognition of the triphasic pattern after the development of post-operative DI is crucial to prevent severe hypo- and hypernatremia. Although in the rat model discussed above, compression of the pituitary stalk was not sufficient to cause damage to the portal system, thus leaving anterior pituitary function intact, our case suggests that all children with DI after ETV should be screened and monitored for anterior pituitary deficiencies. 

## Figures and Tables

**Table 1:**
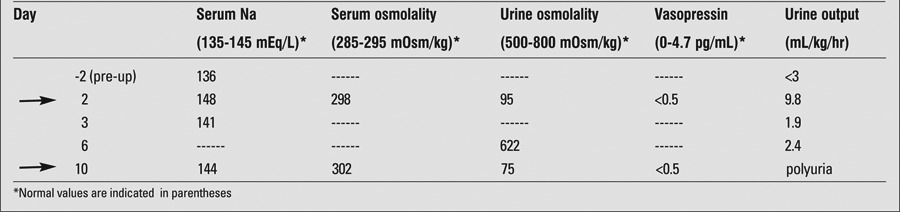
Laboratory evaluation during the clinical course. Arrows indicate time points at which desmopressin was administered

**Table 2:**
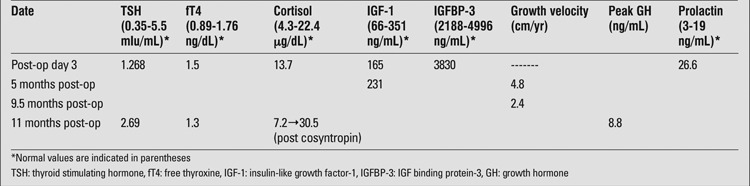
Anterior pituitary function
